# Where to Next for Māori Health Research Review Processes? Insights Into the Indigenous Context: An Integrative Systematic Literature Review

**DOI:** 10.1002/snz2.70058

**Published:** 2026-06-09

**Authors:** Te Hao Apaapa‐Timu, Helen Wihongi, Anneka Anderson, Matire Harwood

**Affiliations:** ^1^ The University of Auckland – Waipapa Taumata Rau Auckland New Zealand; ^2^ Te Whatu Ora ‐ Health New Zealand Auckland and Waitematā Auckland New Zealand

**Keywords:** clinical research, ethics, hospital research, indigenous, kaupapa māori, research appraisal, research consultation, responsiveness

## Abstract

Ensuring that hospital research advances Māori health equity is a key purpose of ethics and locality approval processes in Aotearoa. A systematic integrative literature review method was prescribed, which involved a comprehensive systematic collection and review of database and hand‐searched items using English and te reo Māori terms, MeSH, keywords and questions. A dual appraisal of literature was undertaken using Rayyan software and Microsoft Office, before a descriptive thematic analysis of relevant sources. This article examined 106 literature sources published between 1980 and 2021 to identify and understand how Indigenous review and engagement processes can enhance Māori responsiveness in research. Findings affirmed challenges and opportunities in implementing Māori research reviews, consistent with the pūrākau of Tāne and the attainment of Ngā Kete o te Wānanga. Subsequently, Ngā kete o te Rangahau, the three baskets of research, emerged, generating a framework for considering responsiveness in Māori research reviews.

## 1 Introduction

Aotearoa legislated health research in 1920 and introduced ethical approval in the 1950s through the Medical Research Council, now the Health Research Council of New Zealand (HRC) (Tupara 2011). Under Te Tiriti o Waitangi (Te Tiriti) and the United Nations Declaration of Human Rights and the Rights of Indigenous Peoples (UNDRIP), Māori responsiveness is a constitutional right tied to equity and self‐determination (Côté et al. 2025; Reid et al. 2017). The HRC asserts that all health research in Aotearoa is relevant, acceptable, and accountable to Māori (2010, p. 2; Wyeth et al. 2010). Policies and legislation have since fostered a research environment oriented toward Māori responsiveness, grounded in equal partnership, early engagement, Māori leadership, active participation, Māori epistemologies, and addressing inequities and intergenerational determinants of health (Reid et al. 2017; Smith 1999).

Currently, as part of the ethics approval process, health research conducted in Aotearoa's publicly funded hospitals requires locality approval at each site. Scarcity of Māori consultation in research led to the incorporation of Māori review as one component of the extensive localities approval process (National Ethics Committee 2019) and was administered firstly by Auckland City Hospital (Sporle and Koea 2004). Institutional resistance to resourcing Māori localities review resulted in cultural loading of Māori hospital staff and Iwi partners (Sporle 1999). At the time of this publication, the localities approval process had recently been restructured from a district‐based model to a national, then regional structure, yet still operated as per the former district system, typically involving one‐off consultation, rather than earlier active engagement. The previous health system structure in Aotearoa encompassed 21 District Health Board (DHB) localities (Auckland District Health Board 2008; Loveday and Mitchell 2010), and each DHB had their own localities approval processes and personnel (Simmonds 2015). Inconsistencies across localities approval processes, of which Māori review is one component, caused extensive work for researchers submitting applications across multiple localities (Gear 2020; Loveday and Mitchell 2010). Researchers advocated for a single localities approval pathway across Aotearoa, criticising distinct hospital‐based requirements, some of which were developed to recognise Māori diversity (Stamp et al. 2023).

Māori locality approval is conducted by Māori both informally and in formal capacities, operating within and external to hospitals (Simmonds 2015). Māori locality processes were strengthened when hospital management demonstrated commitment to Iwi relationships and recruitment of Māori research staff (Simmonds 2015; Sporle and Koea 2004). Hospital investment in Māori locality reviews varied regarding resource, administration, personnel, jurisdiction, requirements, management, policy, scope, and ethics (Simmonds 2015; Sporle and Koea 2004).

Efforts to improve Māori localities processes were not forthcoming despite ethical and funding mandates examining Māori responsiveness in health research (National Ethics Committee 2019). The proceeding restructure, nationalising health research remits under Health New Zealand/Te Whatu Ora, anticipated expedited localities processes, including assessment of Maori responsiveness. 3 years into system planning and the restructure, hospital research offices continued to implement separate locality approval processes for each district hospital locality. Trials are underway to nationally standardise general localities processes, including guidelines and Māori application forms, aiming to conduct localities approval regionally, while intending to retain localised Māori review and approval.

**FIGURE 1:**
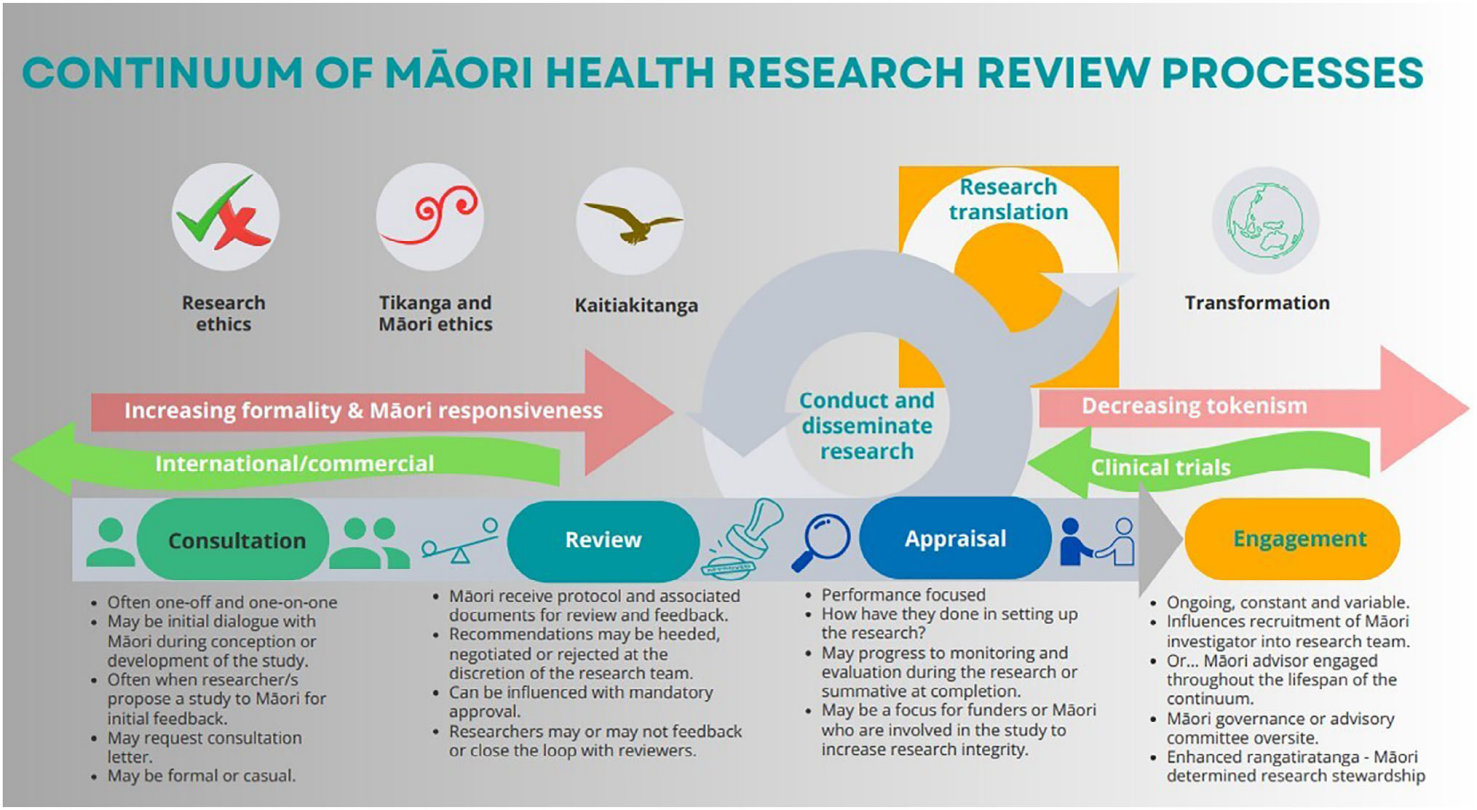
Continuum of Māori health research review processes.

This article reports the findings of a comprehensive synthesis of extant literature regarding the main considerations for robust ethical review of Indigenous health research. Although the experiences of other Indigenous nations in research review processes are pertinent to the scope of this literature review, the results prioritise Aotearoa‐based studies and processes. The scope of literature in this article covers the continuum of research review processes (the continuum) in Figure 1. The continuum is conceptualised as engagements ranging from one‐off consultation with a person identifying as Māori, to formal long‐term Māori governance, advisory, or investigator roles.

The literature frequently points to the appraisal and engaged domains of the continuum as key indicators of best‐practice approaches. Despite recognised standards, current locality review processes in Aotearoa hospitals exclude appraisal, and engagement is limited to a recommendation. Reviewers granting Māori localities authorisation had occasionally advised on the study's development. Resultantly, reviewers approve work that they were involved in developing, presenting inherent conflicts of interest and signalling the need for distinct, independent roles. Notwithstanding the need for role separation, limited resources and the Māori workforce have caused responsibilities to overlap. Maintaining fluidity between roles is important for strengthening research capability and capacity across the continuum, yet must be balanced with clear accountability and management of overlapping functions to ensure translatable and transformative Māori health research. Emphasising distinct roles can help challenge routine understandings that a one‐off consultation or review constitutes genuine appraisal and engagement (Government of Canada 2019).

## 2 Methods

**FIGURE 2:**
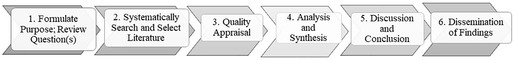
The six steps of the SILR process. *Source:* Toronto and Remington (2020).

A systematic integrative literature review (SILR) of the literature was undertaken, using the systematic approach developed by Cooper (1982) and adapted by Souza et al. (2010) and others (Toronto and Remington 2020; Whittemore, & Knafl 2005) as detailed in Figure 2. The purpose, scope and method of an SILR align better with qualitative methods than other systematic reviews, which focus more on statistical and clinical rigour (Soares et al. 2014; Souza et al. 2010). SILRs integrate studies beyond the scope of quantitative and clinical research, to also include qualitative research (Soares et al. 2014; Toronto and Remington 2020; Whittemore and Knafl 2005).

### 2.1 Step 1: Formulating Review Question(s)

The Patient, Intervention, Comparison and Outcome (PICO) tool has been compared with other frameworks for developing health research questions (Cooke et al. 2012; Methley et al. 2014). Wakefield (2015) supports the adaptation of PICO for qualitative research, coined by theJoanna Briggs Institute (2011) as PICo (Population, Phenomena of Interest, Context). The collective population focus of PICo better aligns with Kaupapa Māori research (KMR) than previous iterations, which focused on individuals. PICo also recognises interwoven phenomenal effects of the social determinants of health, by realising contextual impacts.

KMR influenced the research question by normalising the revitalisation of the social and cultural phenomena of ‘being Māori’, consciously privileging the validation of Māori circumstances, values and realities (Pihama 2010; Smith 2017) and rejecting the homogenisation of Western belief systems (Pihama et al. 2015). KMR provides a platform to articulate and conserve mātauranga in response to the dominant ontological notions of positivist paradigms and Western empirical science (Wilson et al. 2021). Despite three decades of development, there continued to be insufficient mechanisms to ensure Māori authority over research implementation with Māori (Tauri 2014). Maori realities in research entail tokenistic involvement in design, processes and ongoing discrimination and privileging of Western methodologies, knowledge and priorities (Tauri 2014). Marginalisation of Māori knowledge in research exacerbates racism, colonial ideologies and unethical research. Systemic resistance to Māori leadership in health research was a key contributing factor to the extensive health inequities experienced by Māori (Robson and Harris 2007). Substantial progress had occurred, advancing Māori leadership across multiple health research platforms, including genomics guidelines (Abolins‐Thompson et al. 2024) and repositories (Te Aika et al. 2025), data (Greaves et al. 2024; Hudson, Farrar, et al. 2016), tissue sovereignty and biobanking (Hudson, Beaton, et al. 2016b; Hudson, Southey, et al. 2016).

To deter from centralising hegemonic discourse, this article presumes Māori liberation through the research question developed using PICo, ‘Māori health research review processes, where to from here?’

This literature review also attempted to answer additional research questions from the PICo assessment outlined in Table 1:

**TABLE 1:** PICo framework structuring a qualitative health research question.

Population	Māori, Indigenous peoples, health professionals, health researchers, Māori researchers
Phenomena of Interest	Research review processes, health research ethics, and hospital research
Context	Te ao Māori, health settings, clinical settings, Indigenous context

*Adapted from* Joanna Briggs Institute (2011).

1. How should health research be reviewed, appraised or processed to ensure appropriate methods for Māori, other Indigenous and cultural groups who experience marginalisation by other groups in society?
2. What are the barriers to robust research review processes that facilitate culturally appropriate engagement with Māori and Indigenous peoples in health research?
3. What factors encourage researchers to align health research processes with Māori and Indigenous worldviews?

### 2.2 Step 2: Systematic Search and Literature Selection

Inclusion criteria

• Literature between and including the years 1980–2021. The era of the 1980's witnessed a time of reclamation of Kaupapa Māori through the formal establishment of the Kōhanga Reo movement and Kura Kaupapa Māori (Cram 2019; Mahuika 2008) and therefore an important time period to review.
• KMR
• Theoretical and empirical literature
• Quantitative and qualitative studies
• Experimental and non‐experimental research
• International literature
• Unpublished sources
• Grey literature
• Hand‐searched sources
• Literature published in Te Reo Māori and English

Sources referred to one of the following: cultural, ethnic, lore, ethical, moral, value or belief systems and factors that influence the way people participate in or conduct health care research. Sources were relevant to theories and methodologies used to conduct research review processes with Indigenous peoples who had experienced marginalisation by another population. Literature considered the impact of research processes upon populations with Indigenous ancestry or who self‐identified as Indigenous, and groups perceived as marginalised within Western health systems. Literature also focused on how research ideas, protocols and proposals were developed, conducted, reviewed, processed, and implemented.

**FIGURE 3:**
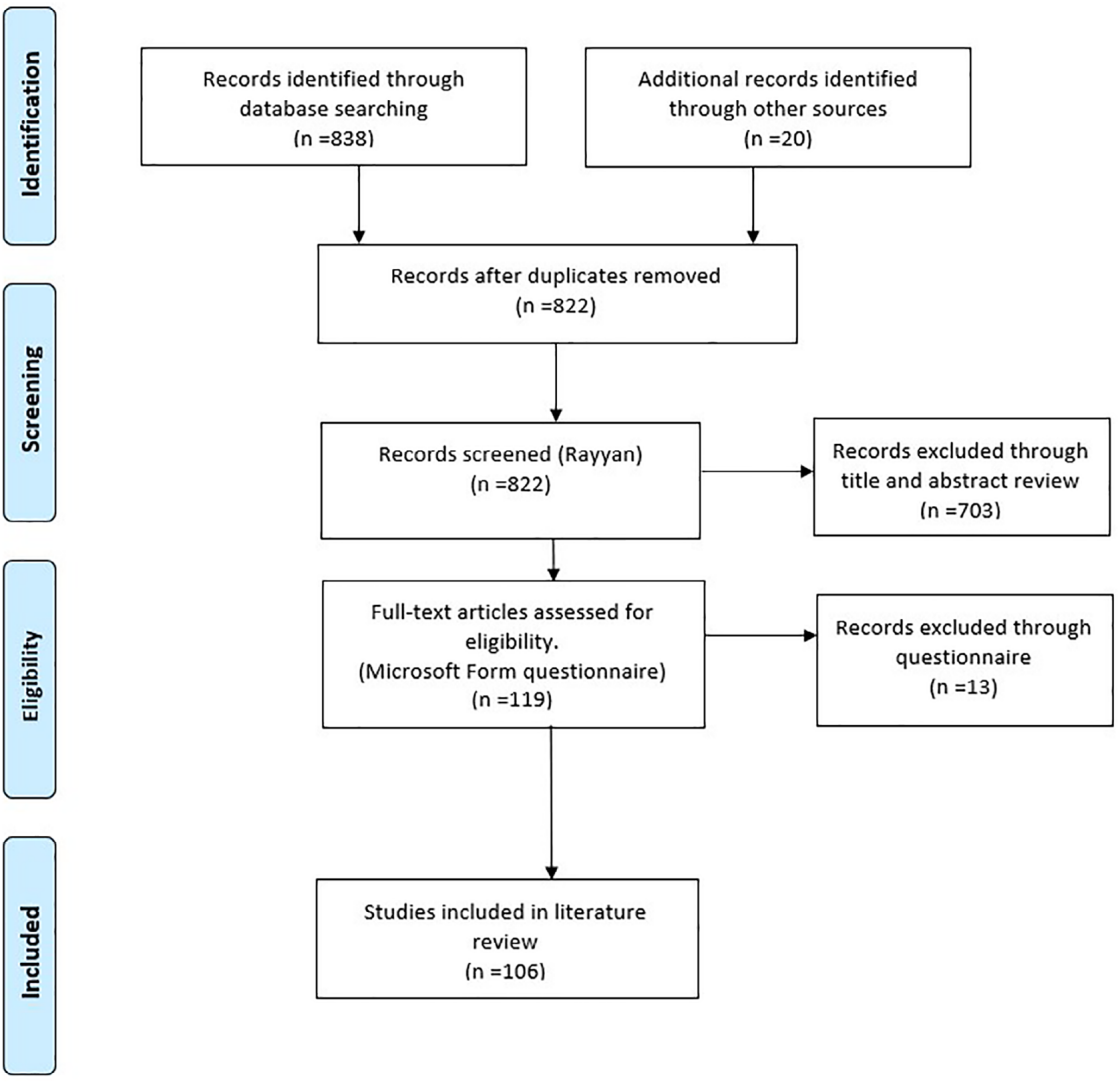
PRISMA flow chart.

In line with IR methods, literature was sourced through two or more approaches, including database, library catalogue and hand searches, minimising bias that may arise from sources that favour clinical research perspectives over broader worldviews (Joanna Briggs Institute 2011; Toronto and Remington 2020; Whittemore and Knafl 2005). Literature searches were conducted using English and te reo Māori terms. The MEDLINE strategy was built using MeSH term hierarchies. Scopus required iterative refinement of keywords, as initial terms produced irrelevant results. PUBMED MeSH combinations generated very large returns, so limiting results to articles with *health* and *research* in the title or abstract improved relevance. Four variations of the research question were run in Google Scholar, and the first 50 results by relevance were appraised. ‘Cited by’ and ‘related articles’ links were recorded, and any missing but relevant items were added as hand‐searched sources in the PRISMA diagram (Figure 3).

Te reo Māori search terms were sourced from ‘Ngā Upoko Tukutuku me Waka Reo’ database (Table S1 Supporting Information). Māori subject headings are especially designed to search within the Australia/New Zealand Reference Centre Plus (ANZRCP) and Te Puna National Library (TPNL) databases. All main concepts were combined with search terms using OR. Two te reo Māori and one English search were conducted in ANZRCP, and one te reo Māori and two English searches were conducted in TPNL. Table S2 in Supporting Information presents how database search terms for the same concepts are combined with OR, then groups of concepts are combined with AND.

### 2.3 Step 3: Quality Appraisal

The internal validity and relevant content of the selected reports were evaluated to determine the strength of the argument and its conclusions (Soares et al. 2014; Souza et al. 2010; Toronto and Remington 2020). The results were exported into EndNote 20 software, dated, and grouped by database. Papers were exported to Rayyan digital systematic literature review software to filter literature by inclusion criteria and identify duplicates. Titles and abstracts were examined to establish an initial inclusion list, followed by a full‐text review where required to confirm inclusion. A Microsoft Office form was used to develop a live questionnaire as a tool to critically appraise the literature based on alignment with inclusion criteria and responsiveness in review processes for Indigenous peoples. The literature was divided between one academic supervisor (HW) and a contracted PhD student for appraisal, the lead author (TA) appraised all the literature for consistency.

### 2.4 Step 4: Analysis and Synthesis

The literature was exported to an Excel spreadsheet to identify the main variables for Indigenous research review processes. The methodological character of the sources contained 30 qualitative, 1 quantitative, 2 metal‐analysis, 1 conference paper, 3 mixed‐method, and 69 literature reviews or viewpoints. TA was used to organise and synthesise recurring themes in alignment with the research questions. In the fourth thematic reflection, the emergent themes aligned with Ngā Kete o te Wānanga (NKotW), the three baskets of knowledge, a Māori story or pakiwaitara Māori. The findings present a qualitative outline of the emergent themes in alignment with NKotW.

## 3 Results

The attributes of the literature relevant to the exploration of this SILR are outlined in Table 2. The sources covered Indigenous peoples from Aotearoa, Australia, China, Japan, Chile, the USA and Canada, Egypt, Turkey, the Pacific, Hawai‘i, and Scandinavia. To address cultural and ethical equity considerations, the criteria also included minority groups such as African Americans, South African communities subjected to internal colonisation by White Afrikaners (1961–1994), and Icelandic populations.

**TABLE 2:** Characteristics of included studies.

Study	Year	Source type	Indigenous population	Indigenous or relevant ethnic co‐authorship
Fetters	1995	Qualitative	Japanese	Yes (Supervisor)
Cox and Macpherson	1996	Qualitative	Caribbean	No
Wallace (1998)	1998	Viewpoint	Iceland Jewish minority population	No
(Kaufert et al.)	1999	Conference paper	First Nations Canada, USA, New Zealand, Greenland and Scandinavia	Yes
(Sporle)	1999	Viewpoint	Indigenous New Zealander/Māori	Yes
(Cunningham)	2000	Viewpoint	Indigenous New Zealander/Māori	Yes
(Paul)	2000	Qualitative	Indigenous New Zealander/Māori	No
(Trotter)	2000	Qualitative	Navajo and General Indigenous populations worldwide	No
(Cunningham et al.)	2003	Viewpoint	Indigenous Australian, New Zealand and Canada	Yes
(Kaufert & Lavoie)	2003	Viewpoint	Indigenous Australian/Aboriginal and Torres Strait	No
(Ermine et al.)	2004	Report	Indigenous people of Canada	Yes
(Hudson)	2004a	Thesis	Indigenous New Zealander/Māori	Yes
(Shambley‐Ebron & Boyle)	2004	Qualitative	African American (minority population in USA)	Yes
(Sporle & Koea)	2004	Viewpoint	Indigenous New Zealander/Māori	Yes
(Dunbar & Scrimgeour)	2005	Literature review	Indigenous Australian/Aboriginal, Koori Peoples	Yes
(Hudson)	2005	Viewpoint	Indigenous New Zealander/Māori	Yes
(Hudson & Ahuriri‐Driscoll)	2005	Conference paper	Indigenous New Zealander/Māori	Yes
(Kingi)	2005	Conference paper	Indigenous New Zealander/Māori	Yes
(Williams)	2005	Viewpoint	African American (minority population in USA)	Yes
(Dunbar & Scrimgeour)	2005	Viewpoint	Indigenous Australian/Aboriginal and Torres Strait	Yes
(Moazam)	2006	Literature review	Indigenous to Pakistan	Yes
(Quigley)	2006	Qualitative case study	Native American	Unclear
(Schrag)	2006	Viewpoint	Native American/Navajo, African American (minority population in USA)	No
(Ahuriri‐Driscoll et al.)	2007	Viewpoint	Indigenous New Zealander/Māori	Yes
(Gifford & Boulton)	2007	Qualitative case study	Indigenous New Zealander/Māori	Yes
(Glass & Kaufert)	2007	Viewpoint	Native North American, Indigenous to Canada	No
(Kidman et al.)	2007	Conference paper	Indigenous New Zealander/Māori	Yes
(Khalil et al.)	2007	Qualitative	Egyptian	Yes
(Street et al.)	2008	Literature review	General Indigenous populations worldwide	Yes
(Hudson)	2009	Viewpoint	Indigenous New Zealander/Māori	Yes
(Hudson & Russell)	2009	Viewpoint	Indigenous New Zealander/Māori	Yes
(Pyett et al.)	2009	Viewpoint	Indigenous Australian/Aboriginal	No
(Shahid et al.)	2009	Viewpoint	Indigenous Australian/Aboriginal	No
(Brief & Illes)	2010	Qualitative	Canadian first nation/Inuit Métis	Unclear
(Cram & Kennedy)	2010	Qualitative	Indigenous New Zealander/Māori	Yes
(Health Research Council of New Zealand)	2010	Guideline	Indigenous New Zealander/Māori	Yes
(Hudson et al.)	2010	Guideline	Indigenous New Zealander/Māori	Yes
(Brunger & Bull)	2011	Qualitative	NunatuKavut formerly Labrador Inuit Métis	Yes
(Koolmatrie)	2011	Qualitative	Indigenous Australian/Aboriginal	Yes
(Maar et al.)	2011	Qualitative	Ontario first nations	Unclear
(Hudson et al.)	2012	Qualitative	Indigenous New Zealander/Māori	Yes
(National Ethics Advisory Committee)	2012	Guideline	Indigenous New Zealander/Māori	No
(Tupara)	2012	Qualitative case study	Indigenous New Zealander/Māori	Yes
(Came)	2013	Qualitative	Indigenous New Zealander/Māori	No
(Chattopadhyay & De Vries)	2013	Viewpoint	General Indigenous and non‐Western populations	No
(Yu et al.)	2013	Qualitative	African American (minority population in USA)	No
(Gubrium et al.)	2014	Viewpoint	North West Alaskan Native	Unclear
(Lawrance et al.)	2014	Qualitative	Indigenous Australian/Aboriginal and Torres Strait	Unclear
(Tauri)	2014	Viewpoint	Indigenous New Zealand/Māori and Indigenous peoples of Canada	Yes
(Gray & Oprescu)	2015	Literature review	Indigenous Australian/Aboriginal	No
(Heffernan et al.)	2015	Qualitative case study	Indigenous Australian/Aboriginal and Torres Strait	Unclear
(Kowal et al.)	2015	Literature review	Indigenous Australian/Aboriginal	No
(Simmonds)	2015	Report	Indigenous New Zealander/Māori	Yes
(Singer et al.)	2015	Qualitative case study	Indigenous Australian/Aboriginal	No
(Tolich)	2015	Viewpoint	Indigenous New Zealander/Māori	Yes
(Brunger & Wall)	2016	Qualitative	Labrador, Southern Inuit	Yes
(Curtis)	2016	Viewpoint	Indigenous New Zealander/Māori	Yes
(Day et al.)	2016	Qualitative case study	Indigenous New Zealander/Māori	No
(Fitzpatrick et al.)	2016	Literature review	General Indigenous populations worldwide	Yes
(Hudson, Beaton, et al.)	2016	Guideline	Indigenous New Zealander/Māori	Yes
(Hudson, Milne, et al.)	2016	Guideline	Indigenous New Zealander/Māori	Yes
(Hudson, Southey, et al.)	2016	Qualitative	Indigenous New Zealander/Māori	Yes
(Robson)	2016	Meta‐analysis	Indigenous New Zealander/Māori	Yes
(Beaton et al.)	2017	Viewpoint	Indigenous New Zealander/Māori	Yes
(Brannelly & Boulton)	2017	Viewpoint	Indigenous New Zealander/Māori	Yes
(Hifnawy et al.)	2017	Mixed method	Egyptian & Lebanese	Yes
(Hiratsuka et al.)	2017	Qualitative case study	Alaskan Native & American Indian	Yes
(Kinchin et al.)	2017	Literature review	Indigenous Australian/Aboriginal and Torres Strait	Yes
(Reid et al.)	2017	Viewpoint	Indigenous New Zealander/Māori	Yes
(Canadian Institutes of Health Research et al.)	2018	Policy Statement	First Nations Canada/Métis and Inuit	Yes
(Ferdinand et al.)	2018	Viewpoint	Chilean	Unclear
(James et al.)	2018	Viewpoint	American Indian Alaska Natives	Yes
(McWhirter & Eckstein)	2018	Viewpoint	Indigenous Australian/Aboriginal	No
(Rosemann & Luo)	2018	Qualitative	Chinese	Yes
(Chandna et al.)	2019	Literature review	Indigenous peoples of Canada, USA, Australia and New Zealand	Yes
(Cram)	2019	Viewpoint	Indigenous New Zealander/Māori	Yes
(Francis et al.)	2019	Qualitative	Indigenous New Zealander/Māori	Yes
(Government of Canada)	2019	Strategic plan	First Nations Canada/Métis and Inuit	Yes
(Hudson et al.)	2019	Qualitative	Indigenous New Zealander/Māori	Yes
(Huria et al.)	2019	Guideline	General Indigenous populations worldwide, with a focus on Māori	Yes
(Keikelame & Swartz)	2019	Qualitative	South African	Yes
(Kukutai & Walter)	2019	Viewpoint	Indigenous New Zealand/Māori and Australian/Aboriginal	Yes
(McPhail‐Bell et al.)	2019	Viewpoint	Indigenous Australian/Aboriginal	Unclear
(National Ethics Committee)	2019	Guideline	Indigenous New Zealander/Māori	Yes
(O’Keefe)	2019	Viewpoint	Native American/American Indian	Yes
(Rix et al.)	2019	Viewpoint	Indigenous Australian/Aboriginal	Yes
(Shih)	2019	Viewpoint	Papua New Guinea	No
(Webster et al.)	2019	Qualitative case study	Indigenous Australian/Aboriginal and Torres Strait	Yes
(Willows)	2019	Viewpoint	General Indigenous, including Canada, New Zealand and Australia	No
(Wilson)	2019	Viewpoint	Indigenous New Zealander/Māori	Yes
(Bull et al.)	2020	Qualitative	NunatuKavut Inuit, Mi’kmaq, New Foundland and Labrador Inuit	Yes
(Burnett‐Hartman et al.)	2020	Quantitative ‐ check	Pacific, Native Hawaiian, Hispanic	Unclear
(George & Tauri)	2020	Viewpoint	Indigenous New Zealander/Māori	Yes
(Harfield et al.)	2020	Qualitative	Indigenous Australian/Aboriginal and Torres Strait	Yes
(Hedges et al.)	2020	Qualitative	Indigenous Australian/Aboriginal	Yes
(Kuzmochka)	2020	Guideline	First Nations Canada/New Foundland and Labrador Inuit	No
(Lin et al.)	2020	Literature review	Indigenous peoples of Canada, USA, Australia and New Zealand	Yes
(Love & Hall)	2020	Viewpoint	Indigenous New Zealander/Māori	Yes
(Rahiri et al.)	2020	Guideline	Indigenous New Zealander/Māori	Yes
(Rasmus et al.)	2020	Viewpoint	Native American & Hawaiian, Native Alaskan	Yes
(Griffiths et al.)	2021	Literature review	General Indigenous populations worldwide	Yes
(Hayward et al.)	2021	Scoping review	First Nations Canada, Métis, Inuit	Yes
(Lovo et al.)	2021	Literature review	Indigenous from Australia, Guam, New Zealand, Pacific Islands	Unclear
(Memon et al.)	2021	Qualitative	Indigenous to Pakistan	Yes
(Murphy et al.)	2021	Literature review	Indigenous Atlantic Canada	No
(White et al.)	2021	Literature review	Indigenous Atlantic Canada and Labrador	Yes

### 3.1 Mai ngā Kete o Te wānanga, Ki Ngā Kete o Te Rangahau – From the Baskets of Knowledge, Emerged the Baskets of Research

**FIGURE 4:**
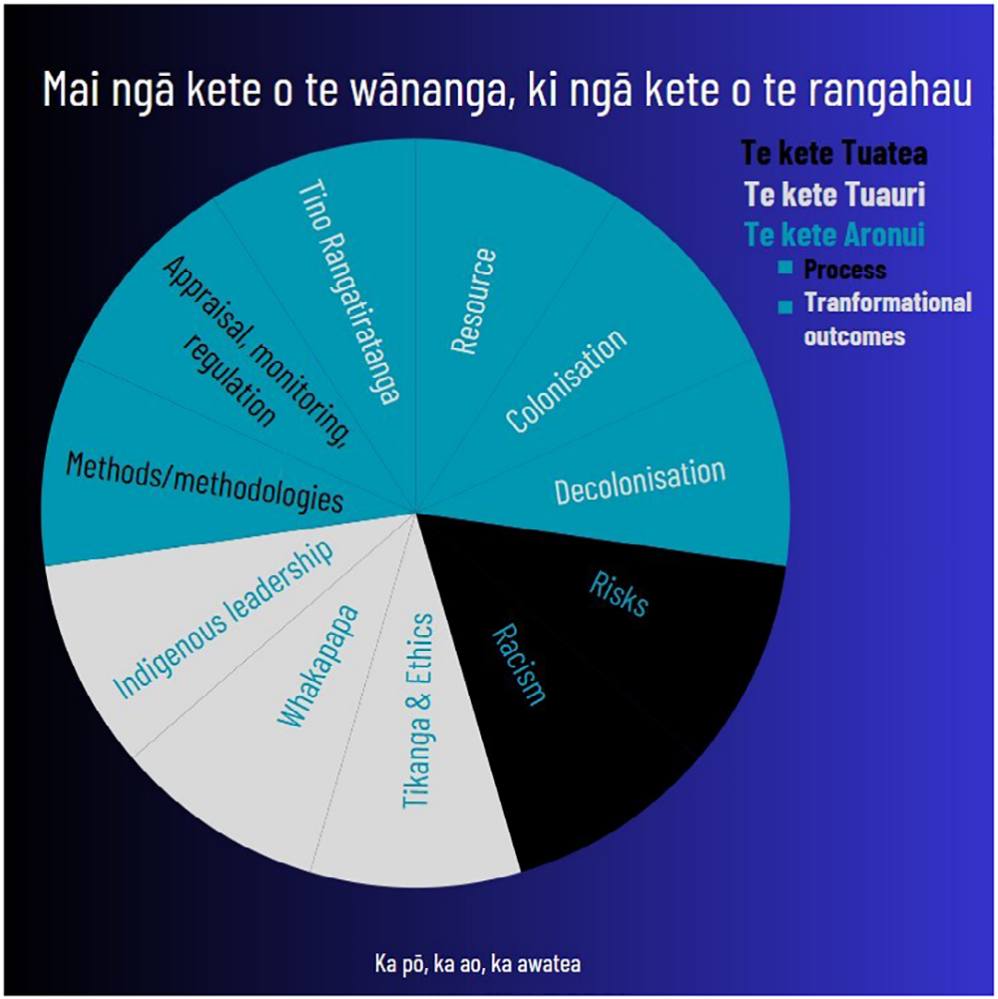
Ngā Kete o te Rangahau (NKotR) – The baskets of research.

According to Māori traditions, Tāne is the God of the forests and their rich ecosystem, distinguished because he successfully separated his parents, Papatuānuku (earth mother) and Ranginui (sky father), after former attempts by his siblings (Reina and Sullivan 1997). Tāne takes many forms; one of these is Tāne te Wānanga, as he skilfully traversed the twelve heavens and acquired Ngā Kete o te Wānanga (NKotW) – the baskets of knowledge (Buck 1949). Traditionally, NKoTW are said to contain all necessary knowledge recognisable to humankind. The results will explain correlations between the emergent themes shown in Figure 4 and NKotW, resulting in sub‐themes, Ngā Kete o te Rangahau (NKotR) – The baskets of research. Mai NKotW, i puta mai i NKotR – From the baskets of knowledge, emerged the baskets of research, a framework for considering Māori research review processes.

The three baskets of knowledge are Te Kete Tuauri – the basket of light; Te Kete Tuatea – the basket of darkness; and Te Kete Aronui – the basket of pursuit, which will be described in sequence to comprise the results of this literature review. Colonisation thematically extended across all kete, as it was relevant throughout Māori and Indigenous research review frameworks, while mostly correlating with Te Kete Tuatea, which encompasses harmful knowledge.

Te Kete Tuauri – the basket of light, encompasses teachings regarding traditional spiritual knowledge, physical energy and guidance (Best 1924). It acknowledges the connection between spiritual and physical phenomena and the protection of the natural world. This kete comprises intangible occurrences, sacred activities, incantation, guardianship and leadership (Best 1924). The three themes most aligned with Te Kete Tuauri encompassed Indigenous leadership – including guardianship, whakapapa – connection, and tikanga and ethics – protection, sacred and spiritual activities Figure 4.

Literature illustrated how Indigenous research leadership is ethical and requires values consistent with tribal contexts (Cram and Kennedy 2010; Gifford and Boulton 2007; Hudson and Russell 2009; Keikelame and Swartz 2019; Lovo et al. 2021; Maar et al. 2011; National Ethics Committee 2019; Tupara 2012). Collective conceptions of the world were a common experience of indigeneity, as opposed to individualistic (Cram and Kennedy 2010; Hudson and Russell 2009; Hudson and Russell 2009; Tupara 2012). Kinship and tribal connections are evident and contextualised in research leadership through ideologies such as: “By Māori, for Māori”; legitimisation of collective whānau and tribal consent; and elder leadership (Came 2013; Ferdinand et al. 2018; Koolmatrie 2011; Memon et al. 2021; Singer et al. 2015). Evidence highlighted the ongoing collective pursuit for Indigenous leadership and self‐determination through active policies to influence the direction, design, management, access and use of research. Iwi such as Ngāti Pōrou and Ngāi Tahu developed tools to collectively assess the value and conduct of research in tribal regions (Reid et al. 2017). Protection was also exemplified by Canada's Mi’kmaq community and Māori Iwi, Ngāti Hauiti, who developed protocols guaranteeing that intellectual property (IP) generated by research conducted in their region remained their property and was appropriately managed and disseminated (Gifford and Boulton 2007). Similarly, Indigenous people from Yukon First Nation settlement lands had regulative authority, requiring researchers to acquire approval to conduct research locally (Ermine et al. 2004).

The genesis and purpose of research is informed by connections – whakapapa, the relative physical and spiritual relationships, their progression and sustainability, between people, taonga (Indigenous data, tissue, genetic and genomic materials), and the environment. The literature contended that research ethics should require more than community engagement for research approval. The literature consistently examined the ways research ethics systems diminished whakapapa by reinforcing privilege for non‐Indigenous people, and structures that sustain colonial relationships of domination in research.

The integrity of research whakapapa was contingent upon the durability of relationships beyond dissemination and the extent of Indigenous control exercised through governance and non‐Indigenous relinquishment of power and domination (Ferdinand et al. 2018; Hudson, Beaton, et al. 2016a). The literature demonstrated that restoring power imbalances becomes possible when whakapapa, expressed through kaitiakitanga (Māori protectorship), is strengthened in the governance of the project. The literature maintained that Indigenous research governance should include the responsibility of caring for venerable taonga, particularly tissue, as people's inherent connection to the environment, recognising that carelessness can inflict spiritual and cultural harm (McPhail‐Bell et al. 2019; Reid et al. 2017; Tupara 2012). Considerations included collaborative biobanks and networks supporting transparency regarding sharing, spiritual connections, sending and return or disposals of taonga (Beaton et al. 2017; Hudson 2004b; Hudson, Beaton, et al. 2016a; Hudson, Southey, et al. 2016). Other tools navigated Indigenous data interests, rights, collection, analysis, interpretation, storage, security, sharing, withdrawal, disposal, return for review and dissemination (Griffiths et al. 2021; Hudson, Beaton, et al. 2016a; Keikelame and Swartz 2019; Khalil et al. 2007).

Few papers endorsed whakapapa through Indigenous regulatory rights promoting welfare, health, and wellbeing (Burnett‐Hartman et al. 2020; Hudson, Southey, et al. 2016). Research reviews and consultation stimulate regulatory activity, often representing the only opportunity for Indigenous connection, contribution, and influence over research (George and Tauri 2020). Despite Indigenous aspirations for enhanced whakapapa in research, requests for responsiveness were often perceived by non‐Indigenous as a procedural barrier and nuisance (George and Tauri 2020; Tolich 2015). Undeterred by researcher resistance, whakapapa was evidenced in the literature as a measure of research integrity in practice.

Tikanga and ethics are pertinent to the realisation of Māori health research aspirations and, when outlined in protocols and recommended in Māori consultation and reviews, should be translated into practice. However, following recommendations from Māori consultation and review, requests were frequently disregarded by researchers (Fitzpatrick et al. 2016; George and Tauri 2020; Gray and Oprescu 2015; Rahiri et al. 2020). Tikanga epitomises whakapapa, Māori language, and cultural values, beliefs, and worldviews, offering relevant, dynamic, safe, and transformational ethical standards for enhancing research (Gifford and Boulton 2007; Hudson and Ahuriri‐Driscoll 2005; Hudson, Beaton, et al. 2016a; Hudson et al. 2010).

As a measure of cultural responsiveness, Māori consultation is a mandatory condition for all research ethics and localities approvals in Aotearoa, usually evidenced with an approval letter (Sporle 1999). Poor tikanga and ethical responsiveness persisted, with minimal Māori representation on ethics committees (Sporle and Koea 2004). Siloed practices intensified Māori cultural isolation, and were frequently observed across locality approval spaces (Sporle 1999). Similar experiences for other Indigenous peoples, gave rise to independent research ethics committees, some involving accountability mechanisms (Dunbar and Scrimgeour 2006; Hiratsuka et al. 2017; Hudson 2004b; Kaufert and Lavoie 2003; National Ethics Committee 2019).

In Aotearoa, Māori consultations for tikanga and ethics are enacted variously, in funded and unfunded private and public sectors, by reviewers, advisors, trusted experts, consultancy firms, governance, community, Indigenous research and tribal committees. The literature suggested that appointments to Indigenous reviewer roles commonly assume that Indigenous ethnicity signified the capacity to enact relevant responsiveness and contribute ethically through cultural values and perspectives. Also, variations in the scope of work are not distinguished, where Māori research review is used synonymously with consultation and engagement, yet all inform ethics or funding processes, and are inherently grounded in tikanga. Indigenous values and ethics were best upheld when Indigenous involvement was prioritised from conception, and throughout a study, affirming the value of Indigenous wisdom and determination (Brunger and Wall 2016; Hudson, Beaton, et al. 2016a; Singer et al. 2015). Progressive research recognises cultural indifference and relevance in science, actions in obstruction to this are not only unethical, but denies human advancement (Hudson et al. 2010). Māori research reviews that promote Indigenous leadership, whakapapa, tikanga and ethics, enable human advancement, protection and mitigation of harm.

Te Kete Tuatea – the basket of darkness, preserves maleficent practices (Royal 2009), including ancient knowledge of harm, both those inflicted or experienced (Best 1924; Buck 1949). Literature relevant to Te Kete Tuatea examined Indigenous experiences of hegemony and oppression in health research. Within this kete, two overarching themes emerged across the literature: risks and racism.

The literature identified risks associated with poor application of Indigenous research guidelines. Risks are compounded when guidelines are poorly implemented and when bureaucracy, commercial sponsorship, and implications for participation and interpretation undermine self‐determination. The literature illustrated risks including intentional deception; breaches of sovereignty concerning tissue, data and IP; white privilege; and inequitable sampling methods and infrastructure; factors that collectively contributed to marginalisation, community fatigue, and erosion of trust. The level of risk amplifies in international research, including commercially sponsored studies, because overseas investigators are not held to the same ethical standards regarding Indigenous responsiveness and are often provided exemptions (Government of Canada 2019). Commercial sponsorship in health research is renowned for influencing ownership structures, methods, and dissemination, resulting in biased investigative outcomes (Fabbri et al. 2018; Odierna et al. 2013).

Guidelines provide a key communication mechanism between researchers and reviewers, requiring clarity, integrity, and honourable conduct. The growing implications of precision medicine, particularly the biological assessment of the Māori genome, produce risks associated with kaitiakitanga (Beaton et al. 2017). Despite the imminent risks of non‐adherence to guidelines, Māori encounter a paradox of feeling obligated to participate for the benefit of present and future generations. Thus, for Māori, weighing potential harm against the health of whānau and mokopuna requires greater recognition of white privilege in the academy.

Racism permeates health research review processes, limiting Indigenous ownership, knowledge, priorities and participation while failing to confront the structural drivers of inequity. Came (2013) reported a paucity of non‐Indigenous commitment to addressing racism in health research, resulting in the perpetuation of discriminatory practices. Racism is further entrenched through deficit theorising in research dissemination and translation (Brunger and Wall 2016; Hudson, Beaton, et al. 2016a; National Ethics Committee 2019; Rahiri et al. 2020; Rix et al. 2019; Willows 2019). Contrarities between Indigenous and non‐Indigenous values and knowledge inherently generate stereotypical evidence within research contexts (George and Tauri 2020; Gray and Oprescu 2015; Rahiri et al. 2020). The literature consistently highlighted divergent Indigenous and non‐Indigenous judgments and values concerning research excellence, funding prioritisation, qualifications, rigour, wairua, responsiveness, integrity, reciprocity, survival and protection, and how these differences shape the interpretation of knowledge. Racism in dominant Western research paradigms has compounded Indigenous disadvantage, and neglected the imperatives of Indigenous health equity and community mobilisation (George and Tauri 2020). Notwithstanding this, Indigenous scholars continue advancements in Indigenous research to rebalance and transform the structures that underpin racism, its discourse and broader impacts.

Te Kete Aronui – the basket of pursuit, contains constructive knowledge that is advantageous to humankind (Best 1924; Buck 1949). Knowledge from Te Kete Aronui is attained through observations, literature, and encompasses love, humanities, philosophy, arts and environmental sustainability (Ruru and Nikora 2021). Within this review, it comprised two themes with six sub‐themes: Process (methods and methodologies; appraisal, monitoring, and regulation) and Transformational outcomes (tino rangatiratanga, resources, colonisation, and decolonisation), see Figure 4.

Indigenous research emphasises alignment between research processes and Indigenous methodological foundations (Cram 2019; Cram and Kennedy 2010; Cunningham 2000; Ermine et al. 2004; Keikelame and Swartz 2019; Maar et al. 2011). The literature highlighted efforts to move beyond prescriptive Western methods toward safe, innovative, and beneficial research, while continuing to address harms associated with Western methodologies, particularly in emerging areas such as genomic research (Beaton et al. 2017; Burnett‐Hartman et al. 2020; Hudson, Beaton, et al. 2016a; Hudson, Southey, et al. 2016). Some non‐Indigenous researchers pursued partnership and harm mitigation through trust and learning, while others retained authority over potentially harmful investigations (Cram 2019; Dunbar and Scrimgeour 2005; Fitzpatrick et al. 2016; Griffiths et al. 2021; Hudson and Ahuriri‐Driscoll 2005; Keikelame and Swartz 2019). Indigenous scholars call for methodologies that recognise moral obligations to return benefits to the holders of Indigenous knowledge and their descendants.

KMR, research conducted ‘by Māori, for Māori’, prioritises Māori worldviews, language, and practices, establishing accountability and standards for research with Māori (Smith 1999; Smith et al. 2016). Participatory Action Research (PAR) similarly supports decolonising research by challenging Western positivist paradigms (Ermine et al. 2004; Memon et al. 2021). Together, these approaches create space for critical dialogue and knowledge construction that privilege Indigenous voice and sovereignty. Cunningham's continuum described four levels of Māori participation: research excluding Māori, including Māori, Māori‐centred research, and KMR (Cunningham 2000). The literature emphasised translating these methodological principles into tangible practice to strengthen Indigenous responsiveness.

There was a propensity for processes that appraise, monitor and regulate research with Indigenous peoples. Scholars called for stronger reporting frameworks and Indigenous‐led indicators to evaluate research quality (Ferdinand et al. 2018; Gray and Oprescu 2015; Hedges et al. 2020; Hifnawy et al. 2017; Hiratsuka et al. 2017; Huria et al. 2019; Khalil et al. 2007; Reid et al. 2017; Tolich 2015). Tools such as the Aboriginal and Torres Strait Islander Quality Appraisal Tool, the CONSIDER statement, and He Pikinga Waiora provided more robust evaluations of Indigenous research quality and outcomes than conventional frameworks (Harfield et al. 2020; Huria et al. 2019; Oetzel et al. 2020). However, Indigenous regulatory mandates often remain limited or fragmented due to power imbalances between communities and institutional authorities (Hudson and Russell 2009; Hudson, Beaton, et al. 2016a; Kaufert and Lavoie 2003; Paul 2000). Strengthening these mechanisms may enable greater collaboration and accountability in assessing research quality.

As Indigenous research leadership advances, so too do the demands for research translation and transformational outcomes. Despite ongoing Western prejudice and infringements, Indigenous scholarship and leadership continue to advance innovative and evidence‐based research (Brannelly and Boulton 2017; Cram 2019; Ermine et al. 2004; White et al. 2021). Strategically, Tino rangatiratanga (TR) represents Māori self‐determination and the discourse advancing its realisation. As a right inherent to Māori as Indigenous people, TR is supported, though not defined, by Te Tiriti o Waitangi (Hudson and Russell 2009). Comparable assertions of Indigenous research sovereignty include the Canadian First Nations principles of Ownership, Control, Access and Possession (OCAP) (Ermine et al. 2004). The literature positioned Māori responsiveness as a precursor for TR. Huria et al. (2019) illustrated how the CONSIDER statement consolidated outlined eight criteria to operationalise Indigenous self‐determination and assess Indigenous responsiveness in research: governance, prioritisation, relationships, methods, participation, capacity, analysis and interpretation, and dissemination. Effective implementation of such frameworks requires sustained investment in Indigenous research infrastructure.

Equitable access to research resources and outcomes for Indigenous peoples is frequently inadequate (Cram 2019; Dunbar and Scrimgeour 2005; Hudson and Russell 2009; Hudson and Ahuriri‐Driscoll 2005; Keikelame and Swartz 2019). Authors call for funding criteria that prioritise Māori advancement and the elimination of inequities (Cunningham 2000; Gifford and Boulton 2007; Health Research Council of New Zealand 2010; Huria et al. 2019), later reflected in the Health Research Council's 2019 implementation of the national health research strategy (Health Research Council of New Zealand, Ministry of Health, & Ministry of Business Innovation & Employment 2019). Despite significant Indigenous investment in safe and effective research, communities remained under‐resourced while benefits often accrue to non‐Indigenous researchers (Glass and Kaufert 2007; Hudson and Ahuriri‐Driscoll 2005; Sporle 1999). Transparency around funding, project performance, research sites, and Indigenous responsiveness was also limited (Cunningham 2000; Cunningham et al. 2003). While the HRC Māori health advancement guidelines promote accountable, transparent, and incentivised research partnerships with iwi, hapū, and Māori communities (Health Research Council of New Zealand, Ministry of Health, & Ministry of Business Innovation & Employment 2019), these expectations can intensify pressure on already limited Māori research capacity when not matched by an expansion of the Māori reviewer workforce. Without additional reviewers, the increased demand for Māori responsiveness risks intensifying workloads and contributing to burnout (Brunger and Wall 2016; Cunningham et al. 2003; Dunbar and Scrimgeour 2005; Simmonds 2015; Singer et al. 2015).

There is consensus that Māori workforce development is urgently needed to strengthen health research quality amid increasing demand and technical complexity. Demand for Māori researchers creates opportunities but also retention challenges due to salary disparities between public and community research sectors (Cunningham et al. 2003; Simmonds 2015; Singer et al. 2015). Efforts to address workforce shortages sometimes compromise research quality or Māori review (National Ethics Committee 2019) and contribute to cultural loading (Brunger and Wall 2016; Singer et al. 2015; Sporle 1999). Reviewers transitioning from other sectors require training in health research fields and in colonisation and its impacts on equity (Gifford and Boulton 2007; Griffiths et al. 2021; Keikelame and Swartz 2019), while Indigenous lay reviewers require support to navigate both Western and Māori knowledge systems (Gifford and Boulton 2007; Hudson, Milne, et al. 2016; Hudson and Ahuriri‐Driscoll 2005; Paul 2000). As one Māori ethics committee member noted, ‘We need more Māori who are briefed, trained and updated. The process for getting new people sounds haphazard’ (Paul 2000, pp.212). Intersectoral collaboration may strengthen research capacity and capability while addressing social determinants of health.

Te Kete Aronui signalled a shift from resistance to revitalisation through the framing of colonisation and decolonisation. Colonisation emerged across all kete and was linked to disrupted intergenerational knowledge transfer, Western‐centric health systems, and research that marginalised Indigenous ownership, priorities, and participation while obscuring structural drivers of inequity. Māori communities and ethical review processes strive to address colonial research practices that undermine Māori health in clinical research (Simmonds 2015). Yet Māori ethical review is sometimes viewed as an administrative barrier rather than an obligation grounded in addressing inequities produced by colonisation and white privilege (Came 2013; Tolich 2015). Māori epistemologies are frequently marginalised and not afforded equal legitimacy to Western knowledge systems (Cram 2019; Rahiri et al. 2020), contributing to ongoing distrust of Western research (Hudson 2004b). However, research partnerships have encouraged some non‐Indigenous researchers to move away from colonising approaches, recognising benefits for research integrity and translational outcomes (Came 2013; Came et al. 2020).

Research transformations grounded in decolonisation advance self‐determination and centred systemic analysis of health determinants as acts of resistance to colonisation. ‘Decolonising research involves liberating the ‘captive minds’ of both the colonised and the coloniser from oppressive conditions that silence and marginalise the voices of the colonised’ Chilisa (2012, p. 14, p.14). This drives Indigenous calls for independent ethics committees and registries with clear accountability and foundations in Indigenous epistemologies (Hiratsuka et al. 2017; Kaufert and Lavoie 2003; National Ethics Committee 2019; Simmonds 2015). Observed alliances between Indigenous and non‐Indigenous researchers create space for recognition of Indigenous expertise, and demonstrated greater advancements towards equity (Came 2013; Huria et al. 2019). Evidence suggested that collective self‐reflexivity among researchers helps dismantle colonial structures and mindsets (Ferdinand et al. 2018; Keikelame and Swartz 2019; Rahiri et al. 2020). Decolonisation of research requires explicitly tolerant acknowledgement of colonial prejudice, authority (Came 2013), and an appetite for Indigenising research systems through research review jurisdiction (Ermine et al. 2004). Authors noted that non‐Indigenous alignment with Indigenous worldviews often emerged from what they learned through sustained relationships with Indigenous communities.

## 4 Discussion

This systematic review confirmed that Māori and other Indigenous peoples continue to assert self‐determination in health research, amid ethical risks and systemic challenges. While Māori health research review guidelines, frameworks, and processes evolved, cultural values and priorities remain intact, suggesting that collective efforts are prompting transformation. Resistance to racism and colonial structures, alongside the drive for equity, motivates Indigenous developments in research appraisal, risk management, infrastructure and collaborative research. The findings considered barriers that limit shifts from consultation and review to engagement on the continuum of Māori health research review processes, illustrating the need for broader support and resources. The findings suggest that adequate investment would assist transparent scalability of Indigenous research reviews, improving measures of research ethics, quality and effectiveness.

Realising equitable and translational health outcomes for Māori necessitates participation in global research spaces, alongside a deeper interrogation of the systemic flaws that create barriers for Māori in research processes. Western ethical frameworks are not only embedded in commercial research before extending to Aotearoa, but currently enable the exploitation of Māori data and tissue. Accountabilities between researchers, Māori donors, and tissue and data repositories are ambiguous, necessitating greater specificity in reporting ethical use and stewardship of taonga. International collective action could leverage locality approval processes as a platform for implementation, enhancing processes and accountability for global Māori data and tissue sovereignty in health research. Expanding initiatives beyond government entities supports Te Tiriti responsiveness and could enable Māori leadership, increased buy‐in, and inform policy related to restructuring localities approval. Pooling resources and the potential for stronger mandates could also ease the demands on the already overextended Māori workforce, who are often tasked with negotiating research ethics and tikanga.

Expecting international refinements in ethical practice while failing to address local barriers that limit rigour in precision medicine for Māori creates a clear contradiction. Despite evidence identifying blood quantum as a more accurate standard for genomic research (Hudson, Beaton, et al. 2016a), governments continue to rely on census‐based self‐identification, undermining the validity of ethnic analyses. Adopting ‘Māori descent’ as the primary inclusion criterion would strengthen the accuracy and relevance of health research and support policy settings that uphold accountability to Māori. Such shifts are essential for delivering reliable biological profiling and targeted therapies, and for confronting policies that actively discourage Māori participation. The validity and reliability of hospital research for Māori are compromised when Māori ethics are systemically relegated, placing disproportionate responsibility on a limited Māori reviewer workforce to uphold Māori responsiveness. This localised pattern is further reflected in recent developments like the Integrated Data Infrastructure, interlinking potentially‐identifiable data across government agencies, generating breaches to privacy, Māori data sovereignty and Te Tiriti obligations (Greaves et al. 2024).

Māori continue to experience workforce shortages, discrimination, and reduced salaries, including within Māori‐designated positions and those that pledged equity and cultural respect (Haar and Martin 2022; Haar et al. 2025). Despite Māori contributions to enriching understanding of Māori health and whānau, and a natural ability for connection, hospital staff remain reluctant to support Māori involvement and professional development within research spaces (Barrett et al. 2025). While Māori workforce marginalisation persists, hospital locality reviews represent growing institutional presence of Māori assessment criteria, alongside those applied through HRC with the Māori Health Advancement Criteria and the adoption of Te Ara Tika by and the Health and Disability Ethics Committees. These earlier shifts in policy and institutional alignment have prompted developments in research practice and, consequently, changes in the attentiveness of Māori responsiveness in the literature. Despite the headway, evidence still demonstrates how Crown and non‐Māori priorities from earlier approaches continue to generate barriers for Māori in research, alongside persistent underrepresentation across all levels of hospital research structures (Barrett et al. 2025). Subsequent literature has continued to emphasise the need for a blueprint to operationalise Māori research review processes. Consequently, Māori continue to report avoidance of health research due to poor design, methods and conduct, due to experiences of non‐Māori dictation over tikanga and Māori worldviews (Barrett et al. 2025).

Tools designed to assess equity and the quality of health research involving Māori continue to emerge, including the ‘equity‐focused implementation framework’ (Gustafson et al. 2024). Despite localised infrastructural developments, fundamental issues in international clinical trials persist, posing significant ethical risks to taonga and processes intended to support offshore stewardship (Abolins‐Thompson et al. 2024; Trudgett et al. 2022). International collaborations have the potential to be realised, as illustrated by Abolins‐Thompson et al. (2024), who developed a model for tikanga‐based ethical practice in collaboration between American research institutes and Iwi, involving biomedical research, live tissue transport, and kaitiakitanga. The model clarified best‐practice tikanga, including offering karakia as an opt‐out to ensure spiritual protection for tissue, and Māori accompaniment during overseas transit. Māori data sovereignty discourse increasingly centres on accountability and the application of Māori values in governing Māori data (King et al. 2026), prompting calls for standardised and tailored agreements to ensure effective implementation (Trudgett et al. 2022). Tools and policies should guide locally grounded solutions for widely used hospital research platforms such as RedCap, while aligning with the complexities embedded in NKotR.

Ka pō, ka ao, ka awatea is a whakatauki that signals the movement from uncertainty to clarity, a shift NKotR advances by promoting transparency and helping researcher reviewers position their applications along the research continuum.

## 5 Limitations

This review has several limitations. Few sources focused specifically on Māori localities reviews, creating reliance on broader Indigenous research review literature. Limited visibility of unpublished reports may have led to omissions, alongside prioritising English‐language materials, which introduced cataloguing issues, including duplication from misclassified te reo Māori titles and authors. As a result, some relevant documents may have been omitted.

The review period is now somewhat dated, though the consistency of findings across earlier and later literature supports confidence in the themes identified. This review contributes to policy and practice, particularly as few articles focused on administering reviews of health research and were concerned with guideline development rather than utility and practice. Further research is needed to design and evaluate ethical processes that support high‐quality, transferable health research for Māori, and to understand the value of review mechanisms in strengthening Māori self‐determination. Additional investigation is required to address fragmentation in ethical processes following Māori approval and to clarify distinctions between research governance and advisory structures. Strengthening this understanding will support emerging best practices and enhance Māori accountability within research systems.

## 6 Conclusion

Health research ethics processes in Aotearoa have worked to strengthen Māori responsiveness for nearly three decades. This article underscores the importance of Māori worldviews, leadership, relationships, and knowledge in driving research resilience and challenging the structures that sustain Indigenous health inequities. The literature presented a legacy reflecting the cultural, social, spiritual, and physical harms rooted in colonisation and racism. There is a growing global need for dedicated, trusted structures that uphold Indigenous health research ethics. Rigid commercial research systems, particularly clinical trials, create direct barriers to Māori research responsiveness in Aotearoa. Developing alternative research pathways is essential to support ethical Indigenous participation and ensure Māori benefit from health research. Strong, collaborative, and coordinated contributions are urgently needed to advance Māori self‐determination in health research, consistent with UNDRIP and Te Tiriti o Waitangi.

## Author Contributions

**Te Hao Apaapa‐Timu:** conceptualisation, background, question formation, design, method, investigation, literature review, selection and appraisal, data curation, formal analysis, writing, project administration. **Helen Wihongi:** appraisal of literature, supervision. **Matire Harwood:** supervision, guidance. **Anneka Anderson:** supervision, guidance, editing.

## Funding

This study was supported by Health Research Council of New Zealand (20/1318 and 23/861).

## Conflicts of Interest

The authors declare that they have no known competing financial interests or personal relationships that could have appeared to influence the work reported in this paper.

## Supporting information


**Table S1:** Te reo Māori search terms.
**Table S2:** Database and keyword search terms, hits and included sources. Key: MeSH terms bolded & Keyword terms non‐bolded.
