# Supplementary material for: Where to Next for Māori Health Research Review Processes? Insights Into the Indigenous Context: An Integrative Systematic Literature Review
Source: J R Soc N Z. 2026 Jun 9;56(3):e70058. doi: 10.1002/snz2.70058 (PMC13248837; doi:10.1002/snz2.70058)
Supplement: Supplementary file 1 — Table S1: Te reo Māori search terms. Table S2: Database and keyword search terms, hits and included sources. Key: MeSH terms bolded & Keyword terms non‐bolded. [file SNZ2-56-e70058-s001.pdf]

Supplementary material

Table 1: Te reo Māori search terms

**Concept**	**Search term**
**Hauora**	Aronganui*, Hinengaro*, Hōhipera*, Kaimahi (ora)*, “Mahi tapuhi”*/Nēhi*, Ora*, Tākuta*, Taonga*, Tinana*, Wairua*, Whakamārama*, Whakaora*, Whakapakari*
**Rangahau***	Haumaru*, Kaupapa*, Whakaaro*, Kairapukorero*, Mana*, Manawhakahaere*, Manawaroa*, Māoritanga*, Māramatanga*, Mātauranga*, “Pātaka pukapuka”*, Patapatai*, Pou tarāwaho*, Rangatira*, Reo*, Ritenga*, Taukumekume*, Tohe*, Tōrongapu*, Uiui*, Waihanga, Wānanga*, Whakamārama*, Whakatoi*, Whakatuwhera*
**Māori**	“Iwi taketake”, “Mana Whenua”, Manuhiri*, Maori, Maaori, Mātāwaka, “Tāngata Whenua”, Taurahere*, Whānau*
**Matatika***	Kōkiri*, Tapu*, Tikanga*, “Tirohanga Māori”*, Kawa*, Tōtika

Table 2: Database and keyword search terms, hits and included sources

Key: MeSH terms bolded & Keyword terms non-bolded

Key concept	MEDLINE	Scopus	PUBMED
Health	***Health services, *Hospitals, *Medical staff, Hospital, *Patient participation, *Physicians,*Physician-patient relations, *Primary health care,*Spirituality**<br><br>Health, Doctor*.ab,ti., Health care.ab,ti., Health Consumer*.ab,ti., Patient*.ab,ti.	Health*, Doctor*, Health, Consumer*, Hospital*, Medic*, Patient*, Physician*, "primary care", Practitioner*, Nurs*	**Ambulatory Care, Attitude of Health Personnel, Delivery of Health Care, Healthcare Disparities, Hospitals, Medical Staff, Minority Health, Patient, Acceptance of Health Care, Patients, Physicians, Physician-Patient Relations, Primary Health Care, Quality of Health Care, Quality of Life, Social Determinants of Health, Spiritual Therapies, Health [TIAB], Health**
Research	***Research, *Biomedical research, *Evidence based medicine, *Informed consent, *Health Services Research, *Research design, *Research subjects**<br><br>Research*.ab,ti.	Research*, Method*, study, studies, Review*, "Clinical trial", "data collection", Process*	**Biomedical Research, Clinical Study, Clinical Trials as Topic, Community-Based Participatory Research, Comparative Effectiveness Research, Comparative Study, Evaluation Studies as Topic, Genetic Research, Health Services Research, Human Experimentation, Qualitative Research, Research Design, Research Personnel, Research Subjects, Research[TIAB], Research**
Māori	***Australia, Cultural Diversity,*Indigenous Peoples ,*New Zealand,*Oceanic Ancestry Group,*Research subjects,*Vulnerable populations**	Indigenous Māori*, Maaori*, Aborigin*.mp., native*, Aotearoa, Zealand*, Cultur*	**Alaskan Natives, American Natives, Indigenous Canadians, Indigenous Peoples, New Zealand, Oceanic Ancestry Group, Patient Selection, Population Groups, Race Factors**

	Māori,Aborig*.ab,ti., Indigen*.ab,ti., Maori*.ab,ti., Maaori*.ab,ti., Native*.ab,ti., Aotearoa*.ab,ti., Cultur*.ab,ti.		
Review	***Bioethics,*Bioethical issues,*Ethical Analysis,*Ethical Theory, Ethics Committees,*Ethics Consultation,*Ethics Consultation,*Attitudes,*Ethics**<br><br>Cultural review.ab,ti., Ethic*.mp., Research Consult*.ab,ti., Research review*.ab,ti., Review process*.ab,ti.	Review*, Moral*, Ethic*, Theor*, Philosoph*, Epistemolog*, Framework*, Model*	**Advisory Committees, Bioethics, Clinical Trials Data Monitoring Committees, Cultural Competency, Ethical Review, Ethical Theory, Ethics, Ethics Committees, Ethics Committees, Ethics Consultation, Human Rights, Moral Obligations**
Total hits	221	217	132
Included	24 (added in additional one from UoA library)	12	13 & 1 maybe

Key concept	Te Puna National Library English Search Terms	Te Puna National Library Te Reo Māori Search Terms	Australia/New Zealand Reference Centre Te Reo Māori Search Terms	Australia/New Zealand Reference Centre English Search Terms
Health	Search One:<br>Health*, Clinic*<br><br>Search Two:<br>kw:(Health* OR Medic*) AND kw:clinic*<br>**Search two used the following filters:** NOT kw:Sociology NOT kw:Anthropol* NOT kw:"Social work" NOT kw:crime NOT kw:Curric*	Hōhipera*<br><br>**To remove irrelevant results the following filters were applied:** NOT kw: project NOT kw: Pedagog* NOT kw: Curricul*	Search One:<br>Hōhipera*<br><br>Search Two:<br>Hinengaro* OR Kaimahi (ora)* OR Tinana* OR Whakamārama* OR Whakaora*	**Ambulatory care, Health care delivery, Healthcare disparities, Health services+, Health services, Indigenous, Medical staff, Hospital, MM “Outpatient Service”, “Patients+, Physicians+, MM “Physician-patient relations”, Primary health care, Quality of life, Spirituality (Omaha), Spiritual Healing+, Spiritual**

	NOT kw:Psycholo*			**Well-Being (Iowa NOC)**<br><br>Clinic*ab,ti., Doctor* ab,ti., Health ab,ti., “Health care”.ab,ti, “Health services”.ab,ti., Hospital*ab,ti., “Patient*”.ab,ti., “Spiritual health”ab,ti.
Research	Search One:<br>Research*, Studies, Study<br><br>Search Two:<br>kw:(Research*)	Mātauranga*, Uiui*, Wānanga*	Search One:<br>Kaupapa*, Mātauranga*, Uiui*, Wānanga*<br><br>Search Two:<br>Kairapukorero* OR Mana* OR Manawhakahaere* OR Manawaroa* OR Māoritanga* OR Māramatanga* OR “Pātaka pukapuka”* OR “Pou tarāwaho”* OR Rangatira* OR Reo* OR Ritenga* OR Taukumekume* OR Tohe* OR Waihanga OR Whakamārama* OR Whakatoi* OR Whakatuwhera*	**Action research, Behavioral research, Clinical research, Consumer Participation, Comparative Studies, Evaluation Research, Experimental studies, Health Services Research, Medical practice Research-based, Nonexperimental studies, Occupational Therapy Practice Research-Based, Physical Therapy Practice Research-Based, Research+, Research methodology+, Research personnel+, Research Subject Recruitment, Researcher-Subject Relations, Research Subject Retention, Study design**<br><br>“Biomedical research”.ab,ti., Research* ab, ti., Study ab,ti.,

				Studies ab,ti., "Clinical Trial*".ab,ti.
Māori	Search One:<br>Māori*, Aborig*, Indigen*, Maori*, Maaori*, Native*<br><br>Search Two:<br>kw:(Māori* OR Maori* OR Maaori* OR Indigen* OR Native* OR Aborig*)	Maori, Maaori, Whānau*	Search One:<br>Maori, Maaori, Whānau*<br><br>Search Two:<br>"Mana Whenua" OR Manuhiri* OR Mātāwaka OR Taurahere*	**Aboriginal Australians, Aboriginal Canadians, Culture+, First Nations of Australia, First Nations of Canada, Indigenous health, Indigenous Peoples, Maori, Native Americans, New Zealand, Research subjects**<br><br>Aotearoa ab,ti., "Indigen*".ab,ti., Māori ab,ti., Aborigin* ab,ti., Native* ab,ti., Aotearoa* ab,ti., "Oceanic Ancestry Group" ab,ti., Cultur* ab,ti., "New Zealand".ab,ti.,
Review	Search One:<br>Review*, "Cultural Review*", Process*, consult*<br><br>Search Two:<br>kw:(Ethic*) AND kw: Cultur*	Kawa*, Tapu*, Tikanga*	Search One:<br>Kawa*, Tapu*, Tikanga*<br><br>Search Two:<br>Tikanga*	**Bioethics, Cultural Competence, Culture +, Cultural safety, Ethics Theory, Ethics+, Ethics Committees, Ethics Consultation, Ethics, Organizational, Process Assessment, (Health Care)+**<br><br>"Ethic*".ab,ti., "Research Consult*".ab,ti., "Scientific integrity review*".ab,ti., "Review method*"ab,ti., "Cultural review*".ab,ti.,

				"Research review*"ab,ti., "Review process*"ab,ti., process*ab,ti., Review*ab,ti.
Total hits	Search One: 23<br>Search Two:24	1	Search One: 10<br>Search Two: 4	6
Included	Search One: 2 maybe<br>Search Two: 4 maybe	1	Search One: 2<br>Search Two: 2	1
Google Scholar questions and search date	Health research with Māori, ethics and process review*<br>Date 21.02.22	Health-related research review processes for Māori* where to from here?<br>Date 21.02.2022	Māori health" research review method*<br>Date 02.03.2022	Health research with Māori/Indigenous peoples, ethics review*<br>Date 18.05.2022
Hits – The first 50 of each appraised	14,700	17,700	38,500	17,800
Included	35	1	1	5

6
